# Prostitutes and criminals: beginnings of eugenics in Croatia in the works of Fran Gundrum from Oriovac (1856-1919)

**DOI:** 10.3325/cmj.2012.53.185

**Published:** 2012-04

**Authors:** Martin Kuhar, Stella Fatović-Ferenčić

**Affiliations:** The Institute for the History and Philosophy of Science, The Division for the History of Medical Sciences of the Croatian Academy of Sciences and Arts, Zagreb, Croatia

## Abstract

Fran Gundrum (1856-1919) was a Croatian physician, encyclopedist, and an advocate of medical enlightenment and healthy lifestyle. In order to identify and analyze Gundrum’s ideas about the problems of prostitution and criminality, we studied all of his books, booklets, and articles published between 1905 and 1914. We showed that Gundrum’s theories of heredity, morality, and sexual hygiene incorporated many of the important discussions of his time, especially those related to the Darwinian paradigm. Gundrum’s project of collecting statistics on prostitutes was the first such study published on the territory of today's Croatia. Although he rejected the notions of born prostitutes and born criminals, defended by Italian criminal anthropologist Cesare Lombroso, he still regarded eugenics as a convenient method of dealing with the ills of society. He believed that criminals were degenerate individuals representing a violent threat to the society and that it was legitimate to use radical means, such as sterilization and deportation, to deal with this problem. Organicistic view of the society prevented him from seeing the individual rights as important as that of the society to protect itself. Nevertheless, this view led to many humanistic ideas, such as the binomial illness/poverty in case of prostitution, which influenced many prominent works of social medicine movement.

Fran Srećko Gundrum (born in Oriovac near Slavonski Brod on October 9, 1856, died in Križevci on July 24, 1919), a Croatian physician and encyclopedist educated in Croatia and Vienna (Figure 1), is described by historiographers of medicine as a forerunner of medical enlightenment and advocate of healthy lifestyle (1). He spent most of his working years as a town physician in Križevci, using primarily preventative methods to treat his fellow citizens. While working as a physician in Bulgaria, where the awareness of the importance of disease prevention was quite low, he developed interest in public health activities and hygiene. As a polyglot he was able to participate in international conferences, where he got acquainted with new ideas and medical advances of the time. He published numerous booklets, manuals, and health guides, focusing primarily on alcoholism, tuberculosis, and dental care, and he was especially concerned about poor psychophysical health of young population (2-9). Some of his works were recommended by the County School Council of the City of Zadar for use in “all elementary schools, preparatory schools, and high schools” and by the Department of Religion and Education of the Royal Land Government in Zagreb “to be purchased for libraries in elementary schools and high schools in Croatia and Slavonia” (10). Gundrum’s book “Tobacco” (11) received a special award by the Paris society *Societe contre l'abus du tabac* in 1903. It should be noted that Gundrum, while working for the Medical Association of Croatia and Slavonia, suggested the development of Code of Ethics for Physicians (12). This idea of his eventually came to fruition in 1901, and again in 1922, when Croatian Medical Association issued the ethical code.

**Figure 1:**
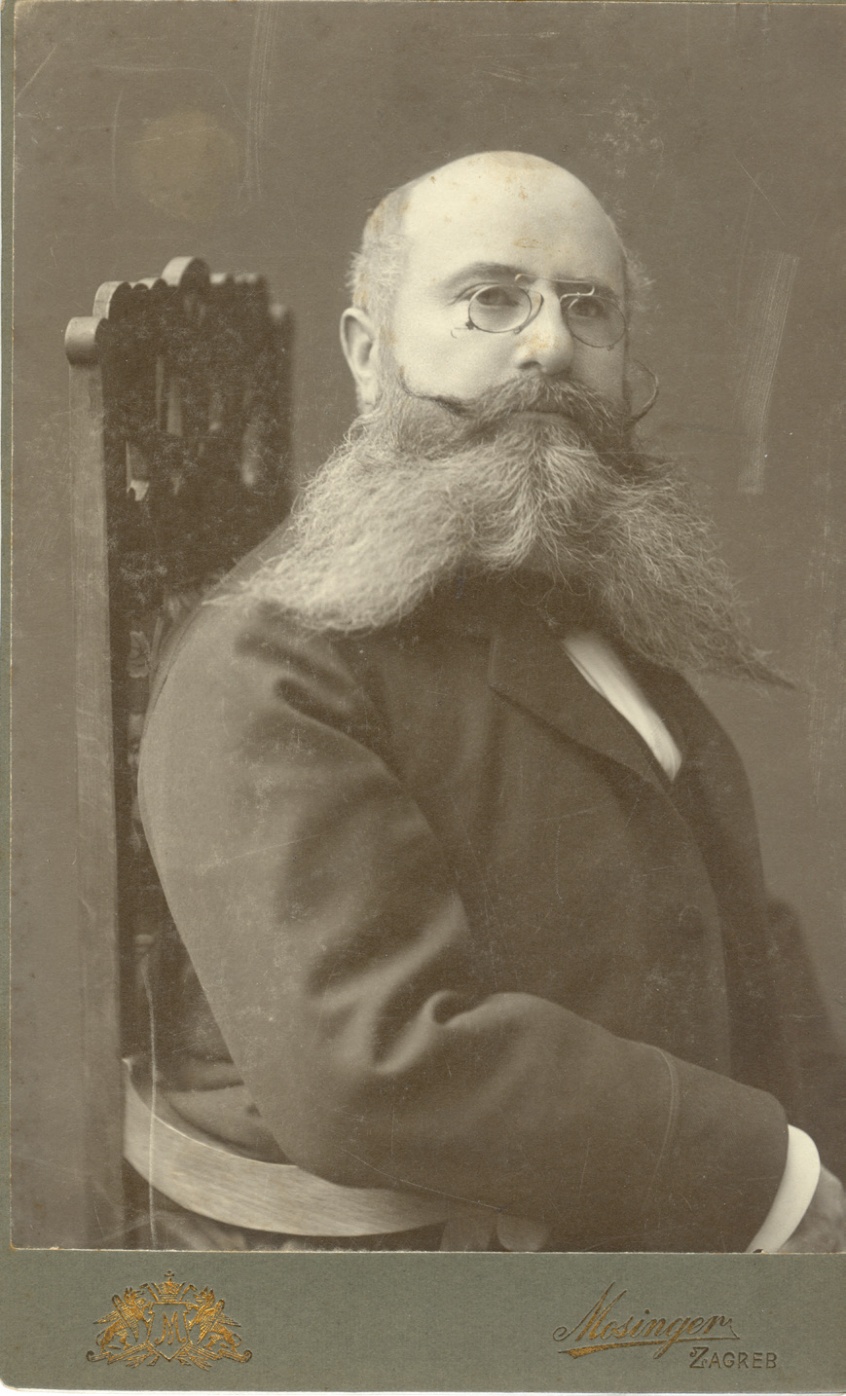
Fran Gundrum (1856-1919). The photograph is kept at the Division for the History of Medical Sciences of the Croatian Academy of Sciences and Arts.

So far, only one comprehensive biographic-bibliographic book about Gundrum has been published (1), as well as several reprints of his works. However, no thorough analysis of his role, influence, and activities has been carried out that would help us determine his place in the history of health enlightenment, eugenics, and other trends at the time in this part of Europe.

We tried to provide evidence that Gundrum’s work on problems of prostitution and criminality contained characteristics of Darwinian ethics combined with characteristics of health enlightenment and occasional elements of negative eugenics, ie, prohibition of reproduction in particular population groups. By providing contemporary theoretical context for Gundrum’s attitudes, we showed that his attitudes reflected the beginnings of eugenic tendencies in Croatia.

We searched through the handwritten material kept at the Department of History of Medical Sciences at the Croatian Academy of Sciences and Arts and all Gundrum’s publications on prostitution and criminality published between 1905 and 1914. In that period, Gundrum was at the height of his professional, intellectual, and writing career. It was also the time when Mendel’s genetics was gaining ground and public health and politics were influenced by the theory of evolution. Many theoreticians of the time argued in favor of Darwinism and saw it as the best paradigm for organization of the society, complementing the Darwinian ideas of natural selection with their moral theories. Thus, the evolutionary ethics was born. Its followers no longer thought that ethical categories of good and evil should be defined according to the Biblical dogmas, but rather to laws of nature (13). The thesis that human beings inherit their moral characteristics just like they inherit their biological instincts offered a different point of view for dealing with ethical issues. The natural selection became a new code for interpretation and evaluation of particular human behavior. An individual was seen merely as a member of a species, and a species as one point in the evolutionary progress (13). The value of an individual corresponded to the value of a cog in the machine. Such a view presented the connection to the organicistic concept of society, which, over time, had a formative influence on attitudes that psychopathology (mental illness, retardation etc.) represented a threat to the society (14). Although Darwin distanced himself from the application of his theory to human society, others like Ernst Haeckel, Alfred Ploetz, and Wilhelm Schallmayer did precisely that. Haeckel, for example, used Darwin’s scientific findings and applied them to social issues, criticizing especially medicine as the main culprit responsible for the creation of a “number of individuals (…) infected by their parents with lingering, hereditary disease” (15).

To convey as accurately as possible the origin, nature, and consistency of Gundrum’s attitudes, we should explain his understanding of heredity, sexual hygiene, and morality. We tried to illustrate how the concepts of sexually transmitted diseases and repression of prostitution were incorporated into Gundrum’s work. We also tried to show how they were incorporated into a broader social context by analyzing his attitudes toward criminals.

## Heredity, sexual hygiene, and morality

In Gundrum’s texts, the concept of “heredity” refers to the “various characteristics of parents and ancestors that can be transferred to their descendants” (16). Among hereditary diseases he includes moles, polydactyly, rotten teeth, shortsightedness, metabolic diseases such as gout, obesity, and diabetes mellitus, mental and neurological diseases, and especially epilepsy (16).

At the time, the basic concepts related to heredity were only started to be understood, and mechanisms of inheritance had not yet been completely explained. Mendel’s laws were rediscovered in 1900, and chromosomes have been known to exist since 1882, but until the seminal work by T. H. Morgan and his associates The Mechanism of Mendelian Heredity in 1915, chromosomes had not been universally accepted as carriers of Mendel’s traits (17,18). Although in 1903 and 1904, Sutton and Boveri proposed that genes were located on chromosomes, genetics still required an independent confirmation that chromosomes could explain specific distribution and combination of traits. Until then, genes had been only seen as hypothesized functional entities, not having a decisive morphological equivalent (19). What finally led Morgan to adopt the position that genes were located on chromosomes and that they were responsible for heredity was the observation that certain traits were sex-linked, together with F. A. Janssens’ cytological demonstration of crossing-over (18). With this knowledge, Morgan constructed the first chromosomal map of the fruit fly *Drosophila melanogaster*. Geneticists in the United States had generally accepted Morgan’s Chromosome Theory of Heredity by 1920, and British geneticists by 1925, while in Germany it had gained acceptance by 1930, but to a lesser extent than in the US or Britain (17). So, Gundrum can very well be excused for maintaining in 1914 that it had not yet been conclusively proven that chromosomes were the sole bearers of heredity. Since his prime concern was the role of external factors in health and disease, he dedicated significantly more attention to blastophtorie. The expression was introduced by Auguste Forel (1848-1931), a Swiss psychiatrist, entomologist, and one of the founders of research into the effects of alcoholism, to show that chronic poisoning of the organism at conception may lead to a serious damage to the fetus, including physical and mental malformations and potential predisposition to harmful social behavior. Because of his views and the belief that weaker members of the society should not be allowed to reproduce, in 1886 Forel himself castrated several mentally ill persons. These are among the first cases of sterilizations reported in Europe (20). Gundrum not only accepted Forel’s blastophtorie, but also embraced the whole theory of inheritance supported by Forel: “(...) each creature [is] an identical repetition of the entire life of its parents or ancestors” (16). In Gundrum’s opinion, the influence of acquired behavior can change genetic material in germinative cells: “Deliberate and abnormal stimuli, which do not originate from the functional maturity of the body, will mediate a significant weakening of the structure through abuse or premature use; weakened testicles will produce a weak and wicked fruit. Thus weakened, the body will produce weak seed and incur suffering to the offspring” (16). One of such abnormal stimuli was alcohol, and it attracted special attention from Gundrum: “Alcohol is harmful not only for the current generation, but also for future generations. It is the cause that prevents normal mental and physical development of the children who come from alcoholics; it is the cause that drunkenness and criminality are inherited and that the descendants of alcoholics also become alcoholics and criminals” (3). These thoughts clearly reflect the influence of Lamarckism, ie, inheritability of acquired characteristics. Jean Baptiste Lamarck (1744-1829) proposed in 1809 the first fully elaborated evolutionary theory, now known as Lamarckism. In his opinion, evolution was a linear change happening slowly over time under the influence of two factors: one was the intrinsic process leading toward ever-greater complexity; and the second was the accumulation of bodily changes that occur with use and disuse of organs (21,22). Those acquired changes were hereditary. Therefore, in his view the role of the environment was key to understanding evolutionary change and the differences found in the living world. Hereditary matter was conceived as soft and moldable. Eventually, Mendel’s genetics contradicted the idea that the environment alters hereditary matter. It is not possible for the protein to make changes to the genes in germ cells, the fact that will later become known as the central dogma of molecular biology (23). Moreover, Darwin’s theory that natural selection operates on variations randomly occurring in each new generation, explained better the phenomena found in the living world. Although Lamarck’s theory of evolution had already given way to Darwin’s theory of natural selection by the time Gundrum wrote his works, Lamarckism was still surviving in various forms. Whether latent or clearly expressed, it was present in the concepts of many physicians of the time, who used it, among other things, to legitimize their public health activities. Socialists especially preferred Lamarckism over Darwinism (24), since they believed that a radical social change could bring about a change in human nature. Gundrum, while certainly not a socialist, also believed that various public health interventions could change human hereditary constitution, which was one of the reasons why he accepted Forel’s Lamarckian notion of blastophtorie, rather than Darwin’s random variations and “hard heredity.”

The idea of hygiene in a socio-medical sense came to life at the end of the 18th century. Its purpose and aims were popularized by a compelling book A System of Complete Medical Police by Johann Peter Frank (1745-1821) (25). The book emphasized that degeneration and illness were the consequences of social inequality, a concept that paved the way for hygiene as a scientific and practical discipline important for the whole society. Gundrum’s work was completely in line with these ideas, especially when promotion of therapeutic power of health education was concerned. Gundrum considered health education to be the basis for prevention and repression of socially harmful diseases. Over the course of the 19th and first half of the 20th century, these ideas were spread and developed further, finding their confirmation in the works of other physicians, such as Andrija Štampar.

**Figure 2:**
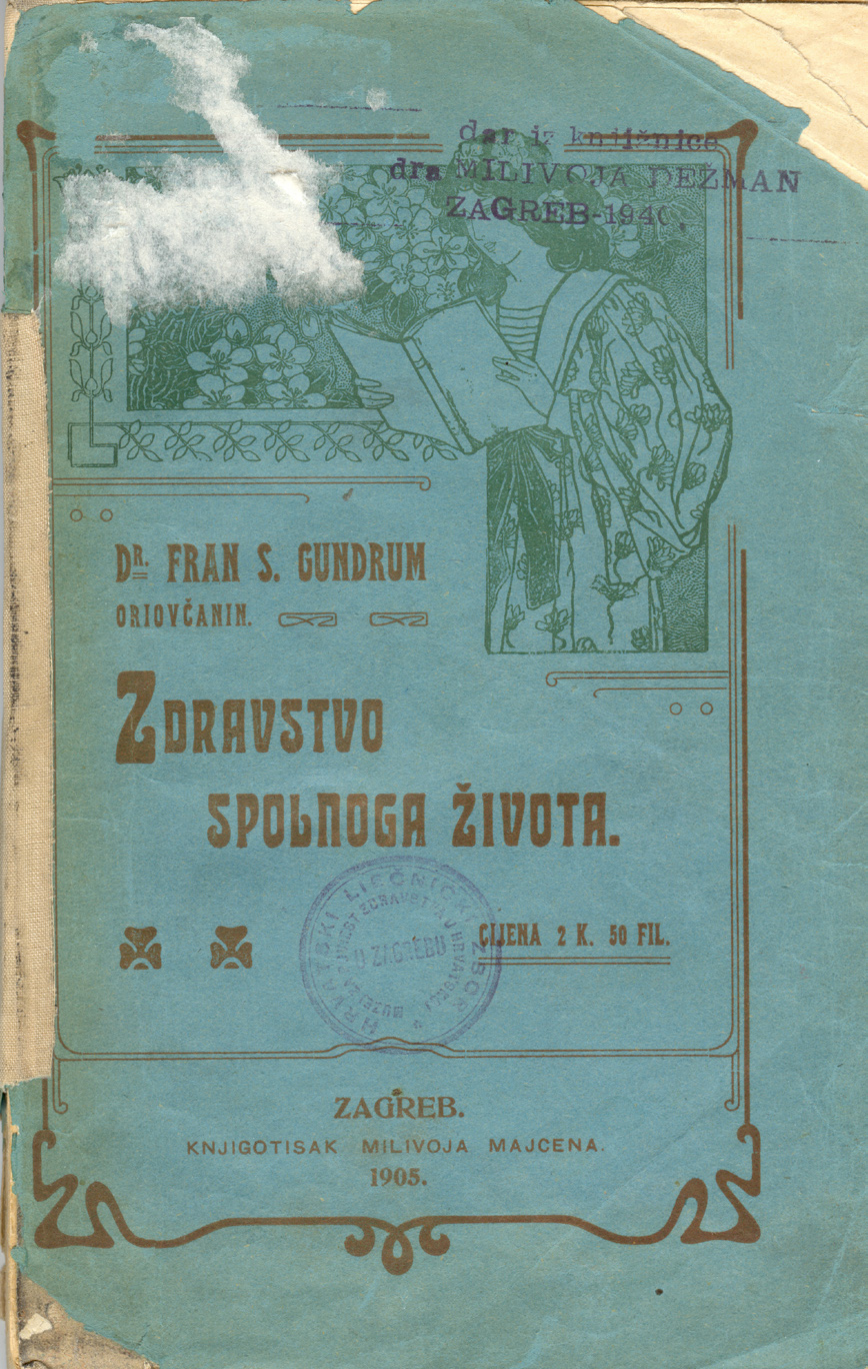
The front cover of the book Sexual Health Care (in Croatian: *Zdravstvo spolnog života*), 1st edition, 1905.

Fran Gundrum was the first physician in the region who was thoroughly devoted to the use of contemporary scientific methodology in his approach to the problems of sexuality. He published his opinions and research results in a comprehensive book, Sexual Health Care (Figure 2) first in 1905 and then again in 1914 as a revised edition (16). It was the first book on sexual health in the region, a textbook type of publication of approximately 500 pages, which investigated all aspects of sexuality, from anatomy and physiology of sexual organs to the causes of spread of sexual diseases. The book covered a wide range of topics. Although some chapters were conservative, doubtlessly reflecting author’s own attitudes, such as “Restraint from Sensual Indulgence,” “The Consequences of Unrestrained Sexual Activity,” or “Repression of Onanism,” overall the book was completely modern in a sense that it openly discussed many topics that were usually not talked about, such as masochism, sadism, and homosexuality. We should also mention that Gundrum self-published the book because he could not find a publisher. In his advertisement for the book, Gundrum says: “Venereal diseases are spreading faster than ever through all layers of society, jeopardizing the health of man and his offspring. It would behoove everyone to learn more about their essence and nature, their origin and development, for the purpose of self-protection. To that end, in a few days’ time, I will be publishing a book written in a popular style, titled Sexual Health Care(...). The book provides ample advice and exhaustive account of almost all facets related to the issue. With respect to its content, Sexual Health Care is the first of its kind in Croatian literature. The time has come to fulfill the urgent need and thoroughly instruct a man on what benefits and what harms his health. Various problems that have been left untouched despite their far-reaching consequences now have to be broached, in the interest of an individual as well as general public. Our duty, our love of our fellow man demands it. I am hopeful that this work of mine will be successful in gradually stopping the dissemination of venereal and other diseases related to sexual drive” (26).

Gundrum considers sexual hygiene very complex and closely related not only to many physiological processes in the body, but also to mental conditions and growth and development of an individual. With respect to its strong influence on every aspect of individual and social life, sexuality was, in Gundrum’s writing, an unavoidable area of (self-)regulation: “And this statement should serve to many as guidance of sorts, to prevent further harm. For can there be any greater shame than a man with a disease marrying, maybe even without knowledge that he can pass it to his children? Is not such a man almost a threat to the mankind, for sowing the seed that cannot but beget another sick and stunted offspring, offspring that is weak in mind and body, offspring that succumbs to illness easily and remains ill for lengthy periods of time, lacking ability to fulfill his duty to mankind as he ought to; such offspring, I am certain, would be the first to say loud and clear that he would have been better off had he never been born in the first place! There is enough misery in the world! Why increase it further?” (16).

This is an interesting, although not atypical concept of morality at a time when morality was only ceasing to be defined according to religious dogma (13). The evolutionary paradigm did not place man above nature, but saw him as part of nature and morality as a naturally inherited trait. Nature and its laws became a new moral authority. Science rather than ethics as a separate discipline became the authority that prescribed moral rules. Gundrum concurred: “This is the purpose of science – in this case, sexual science – and science is nothing but investigation of truth, a clear wellspring of genuine morality and real humanitarianism” (16). The science of sexual hygiene became one of the most important fields in medicine, and since it directly affected future generations, it was also important from the ethical point of view because it established the real morality. Thereby, Gundrum assumed an implicitly critical attitude toward traditional concepts of morality, and found the laws of nature and its sometimes cruel logic more persuasive than socially construed norms. In his text On Banishment of Criminals, Gundrum refers to natural selection as follows:“[Selection] (…) makes sure that anything that does not comply with its purpose, anything that is weak and unnecessary, is removed. A predatory beast whose teeth get rotten, a bird hatched from the egg as an albino and therefore easily spotted; an animal that becomes mentally ill or psychologically abnormal or develops unusual, inappropriate drives, as well as any other animal that would produce degenerated offspring – perishes early and speedily, definitely before its time to reproduce. Therefore, we may conclude that there is nothing degenerate in the nature” (27).

Gundrum continues with the intent to show that allowing the weak to survive is unnatural, but sensible only if such individuals are expected to be somehow useful. Thus, he laid the foundations for his own objections to such politics and social uselessness of the weak: “However, this logic is only applicable to wild animals; we will not exterminate a domesticated, tame beast for a minor defect, but rather we will protect and keep it, in order to have some use of it. If we use such an animal for breeding, though, then we become complicit in the degeneration of the species. Sometimes we even purposefully breed animals with peculiarities, which would otherwise be exterminated by nature immediately. For example, we breed white cattle; or hornless cattle, which would not be able to defend itself in the wilderness; fat pigs, which would perish in the wild as would a skinny race horse, etc. Specific types of degeneration enable us to achieve a particular purpose; we protect and keep such creatures because we are able to benefit and profit from them; but nature does no such thing; nature destroys all that cannot survive in the normal and usual state of things” (27).

A man, especially a physician, tries to save every individual irrespective of the cost of such intervention: “It is our duty to protect every freak of a man, the sickest, the feeblest, the poorest child, because we cannot eliminate them like ancient Greeks eliminated their children. We must use all possible means, which are sometimes very expensive, to help such a poor creature become able to live as it chooses” (27). In Gundrum’s opinion, by saving the weak and degenerate, medicine has an unnatural purpose because it contradicts natural laws and hinders the development of society of higher quality: “These rescued creatures, to use such an expression, are rarely ‘antisocial,’ usually because they are too weak to ever pose a danger to anyone; on the other hand, they still marry when they become adult enough. (...) However, if a spouse of such an individual, who was only saved from nature after a great effort, is hale enough, we are confronted with a truly bizarre and frequent phenomenon of their children being naturally normal, but nevertheless suffering from some defect; and this is the point where the degeneration begins to manifest itself to a lesser or greater extent” (27).

For Gundrum, morality is a “healthy inner state of man,” and health is a “harmonious working of all forces and activities, so that even natural drives and inclinations of a man, ie, selfish elements, are naturally expressed and thus bound to work harmoniously together with altruistic abilities” (16). The idea of moderation has been known since Aristotle’s times, but the definition of moral characteristics as “natural drives and inclinations” unquestionably results from a Darwinian understanding of ethics, which changes along with a biological, evolutionary progression. Morality is not given once and for all, it is not eternal and immutable, but rather changes as the man changes. The attitude that egoism plays a role in morality could not be derived from Christian teachings; however, this attitude was common to many authors at the turn of the 20th century who considered Darwinism as a foundation of ethics. Moral rules were thus considered to be inherited, serving only as an instrument of survival. Such a belief led to an attitude that every change that improves survival is, in a sense, valuable and good. One of the scientists with a similar attitude to that of Gundrum's was Ernst Haeckel. In his book The Riddle of the Universe from 1900, Haeckel says: “[Modern science] regards as the highest aim of all morality the re-establishment of a sound harmony between egoism and altruism, between self-love and the love of one’s neighbor…”, pointing out that Christianity devoted too much effort to altruism, while neglecting self-love (28).

Sexual hygiene was thus associated with heredity, and heredity was associated with morality. A man had to protect himself from sexually transmitted diseases and irresponsible behavior or else he would ruin himself, his children, and eventually the whole nation and state. The collapse of the nation and state would be an inevitable consequence of irresponsible behavior of individuals and of a society that omitted to sanction such a behavior, because the selection mechanism benefits the stronger and destroys the weaker. Since all these processes were thought to be inherited, and science the only way to investigate the mechanisms and consequences of heredity, it followed that science also determined the fortune or misfortune of people. Regulations on what was considered a moral behavior could not be prescribed any longer by a non-natural authority, but only and exclusively by science. Since science could not lie, what it claimed to be true had to become a new behavioral criterion. Only science could provide guidelines for behavior that led to fortune and progress of the society as a whole. The society, thus, turned out to be an entity whose survival and development was given more importance than individual rights, and the moment when the threat to the society was interpreted as large enough, the society had the right to protect itself.

## Gundrum’s survey and the first study of prostitution in northwestern Croatia

**Figure 3:**
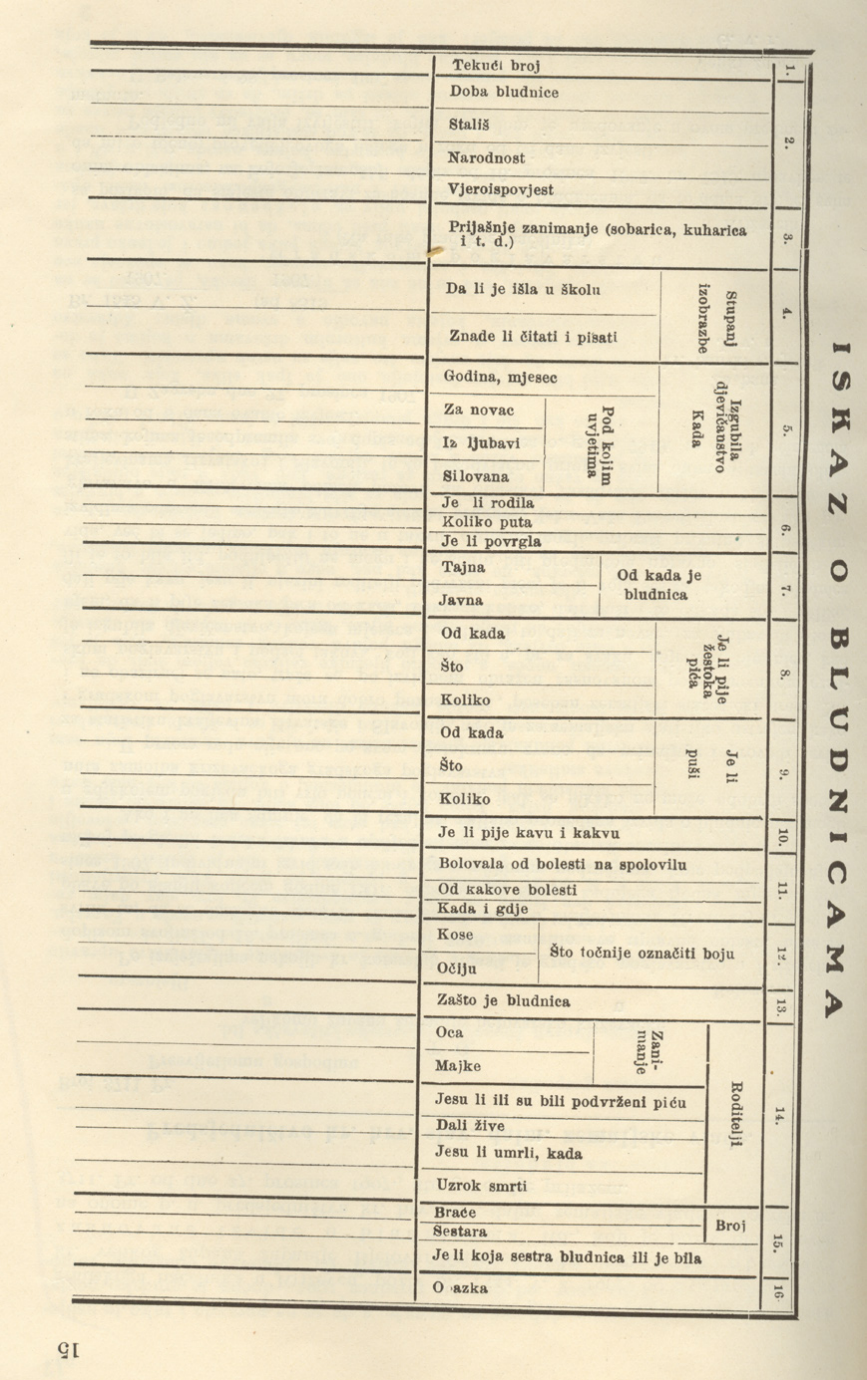
Statement on Prostitutes (in Croatian: Iskaz o bludnicama). From: Public Prostitutes in Croatia and Slavonia in 1907-1908 (in Croatian: *Javne bludnice u Hrvatskoj i Slavoniji 1907./8. godine*), page 15.

In his practice, Gundrum saw a large number of cases of sexually transmitted diseases and gave a lot of thought to how to eradicate them. While he was working as a town physician, in 1907 he started a project of collecting statistics on prostitutes in Croatia and Slavonia in 81 administrative districts (Figure 3). On the basis of 77 replies to his questionnaire, he wrote an extensive article that was published in *Liječnički vjesnik* in 1910 under the title Statistics on Public Prostitutes in Croatia and Slavonia, 1907-1908. Gundrum also gave a lecture based on the results of this project at the General Meeting of the Medical Association of the Kingdom of Croatia and Slavonia. The project material prompted him to revise extensively the first edition of Sexual Health Care, dedicating the largest portion of the book to prostitution. This was the first study and the first publication on the topic of prostitution in Croatia. The basic motivation for this research was Gundrum’s belief that prostitution, a “disgusting infamy” as he called it, was the biggest threat to sexual health of the nation. He reasoned that prostitution brings about debauchery and leads to spreading of venereal diseases. He also believed that detailed and multifaceted statistical research on the problem will reveal the laws that govern this phenomenon, which will enable the society to reduce the negative impact of prostitution. Gundrum highly regarded statistics and even quoted Baron Hugo von Haan’s description of statistics as “a snapshot of life” (16). His survey included questions on prostitutes' age, religion, ethnicity, sexual diseases, reasons for choosing the profession, and many others.

## Gundrum vs Lombroso: “It must be admitted, though, there are some very honest prostitutes.”

Gundrum’s research into prostitution went beyond mere data collection, aiming to illuminate all the intricacies of its causes. His research is not only a scientific study, but also a sort of a cross-sectional review of the ideas of several European authorities in the field of sexuality. It should be noted, for example, that Gundrum had a very different attitude from that of Cesare Lombroso (1836-1909), Italian psychiatrist whose work on antisocial personality typology, which would be abandoned and discarded later on, still attracted a lot of interest at the time. Gundrum criticized Lombroso’s theory about prostitutes, which was essentially an extension of his theory about born criminals. Lombroso considered prostitutes equivalent to criminals and believed that prostitution was a hereditary disease. He also believed that prostitutes could be recognized by physical and psychological flaws. Gundrum considered Lombroso’s theory about women unsound and pretentious, because it concentrated on women's inadequacies compared to men (16). It was clear to Gundrum that a large percentage of prostitutes were in the profession out of economic necessity and due to neglect in childhood, as indicated by his survey. He emphasized that “there are some very honest prostitutes” (16).

**Figure 4:**
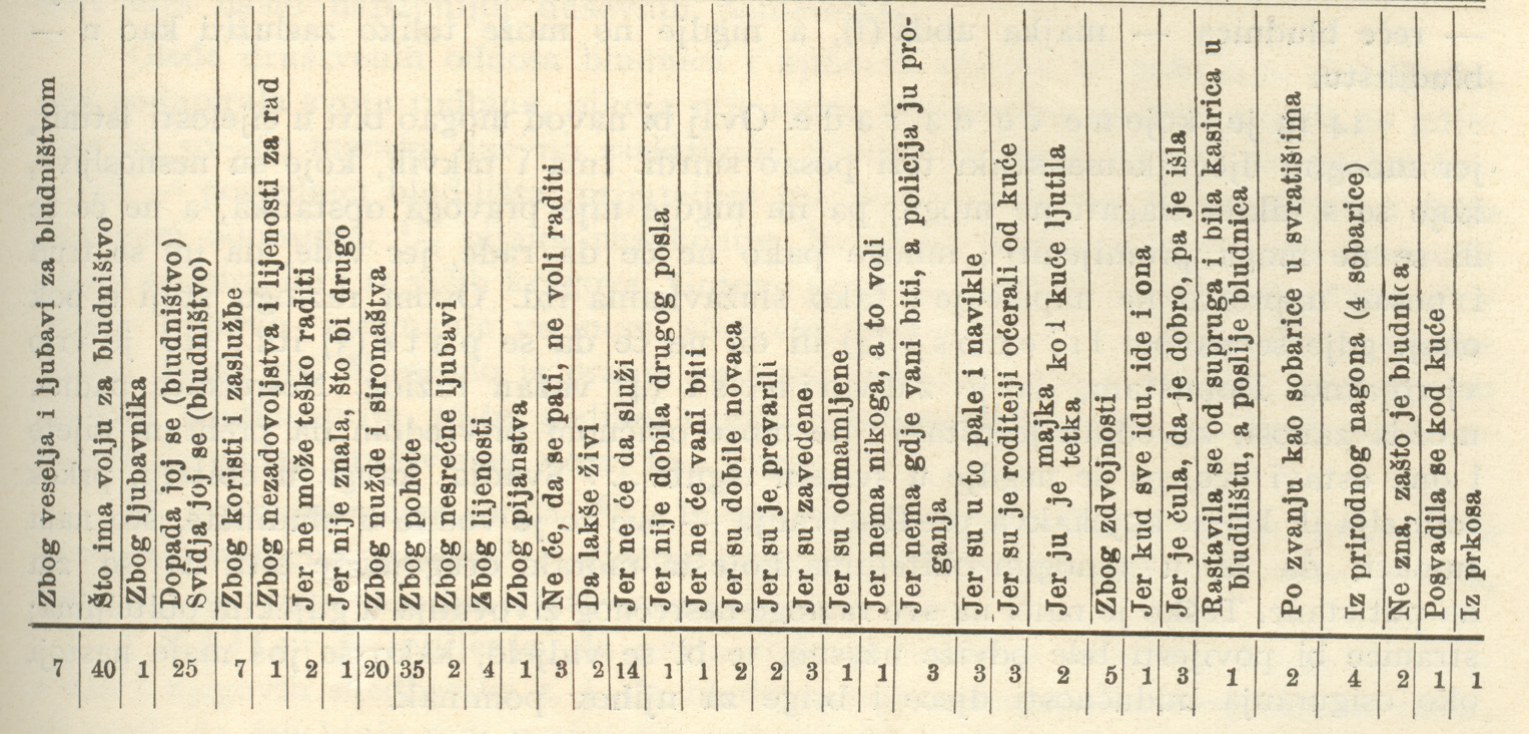
Responses to survey question on the reasons for becoming prostitutes. From: Public Prostitutes in Croatia and Slavonia in 1907-1908 (in Croatian: *Javne bludnice u Hrvatskoj i Slavoniji 1907./8. godine*), page 31.

Among more important issues that caught Gundrum’s attention were those related to intrinsic predisposition that influenced women to turn to prostitution. He obviously did not accept the attitudes of Lombroso, who compared prostitutes to criminals, but instead took a more moderate approach. However, he could not completely resist the idea of an intrinsic predisposition, and the scientific method he used did not convince him otherwise. Of 207 prostitutes who made the core sample, as many as 40 declared they had become prostitutes because of the will for lechery, 7 listed pleasure and love for lechery, 25 stated that they liked it, and 35 listed lust (29) (Figure 4). Based on the replies from half of the subjects, he concluded there was an endogenous cause responsible for their behavioral disorder. By rejecting Lombroso’s concept and by taking into account his own statistical indicators, Gundrum recognized two elementary factors that played a role in prostitution. The first one was upbringing, which was the main reason in most cases: “(...) in a word, the cause could be some mental flaw, which in many cases is the consequence of flawed upbringing and neglect in childhood” (16). The economic milieu was considered only as “the second, contributing factor” (16). Most prostitutes, according to Gundrum, were neither ill nor criminals and “only a few are hereditary degenerative” (16). However, there was a group of prostitutes in whom the cause was hereditary in nature; it did not mean predetermination, but it did imply a certain inclination: “As for the females, it should be said that there are some prostitutes who are degenerate. Nevertheless, this does not mean that they are predetermined to become prostitutes, although it definitely makes them prone to a less stable life. Their nature is riddled with a certain inconstancy; moral, social and other notions have no foothold there. Degeneracy is sometimes manifested in immediate disease, such as severe hysteria or feeblemindedness. Cramps, headache, alcoholism, proneness to fancies, daydreaming and other purposeless practices. Sometimes, the signs of degeneracy are visible in the body, eg, in the shape of the head; in the unusualness, crookedness of the lips – harelip; in the throat – cleft palate; inborn blindness; bulging or eccentric pupil; crookedness of the earlobes, arms or legs; poor development of the entire body; stunted development with deformation of body parts, especially sexual organs; squinting; stuttering” (16).

Whether it was that a girl became a prostitute because of poor upbringing in the early childhood – in majority of cases – or because of the inherited behavioral instability – in minority of cases – neither factor alone could have had such a strong influence, so Gundrum considered social circumstances (eg, poverty) to have an effect on both factors. Thereby, he developed his own theory, according to which, contrary to Lombroso, no prostitute was born as such. However, it is interesting to note that Gundrum classified a possible heredity of inclination toward prostitution together with some stereotypical characteristics of congenital defects, which shows how deeply he was influenced by theories of his time and how much he owed to Lombroso, despite their differences in opinion.

Gundrum’s deep resentment toward prostitution and venereal diseases as its consequence prompted him to clam that syphilis “affects entire social classes and, therefore, sexually transmitted diseases are the public enemy number one.” (10). Despite his opinions and the fact that he considered prostitution to be evil, Gundrum did not condemn prostitutes. He was equally critical toward men who supported prostitution, and especially toward economic system in which young women sometimes had no other choice but to become prostitutes (16).

## Control of prostitution and protective measures

Whichever of these two main causes of prostitution was crucial in individual cases, it did not mean that nothing could be done about prostitution. As far as intrinsic factors were concerned, Gundrum was convinced that prostitutes “can often (...), using an appropriate approach, be brought back to the right path” (16). In his first longer text about prostitution, On Sarajevo brothels, published in *Liječnički vjesnik* in 1903 (30), Gundrum made clear that prostitution was a universal occurrence and that everything should be done to bring its negative consequences under control, but he was also aware that it cannot be eradicated. In Gundrum’s opinion, brothels in Sarajevo could serve as a role model to others (30). They were located in a side street in the outskirts of the city, adjacent to the “examination house.” Gundrum thought that it was mandatory to perform physical examinations of prostitutes twice a week by county town physicians and once a week by a city physician. He suggested to brothel managers to use a method he himself had successfully applied in Bulgaria, ie, to write on a board the numbers of rooms and names of prostitutes along with the results of their examination. If a prostitute had her period, he suggested it also should be posted on the board. In this way, prostitutes would not have to use intravaginal sponges, which were harmful to their health and “deceitful to their guests” (30). In addition, Gundrum suggested reeducation of underage prostitutes, sanctioning women trafficking, reporting sexually transmitted diseases, and mandatory treatment of sexually transmitted diseases. The decisive role in these actions had to be played by the state, which had a free hand to put prostitution under control by systematic and thorough implementation of public health and preventative measures.

## Healthy and sick criminals – Gundrum’s attitudes toward degeneration and deportation of criminals

**Figure 5:**
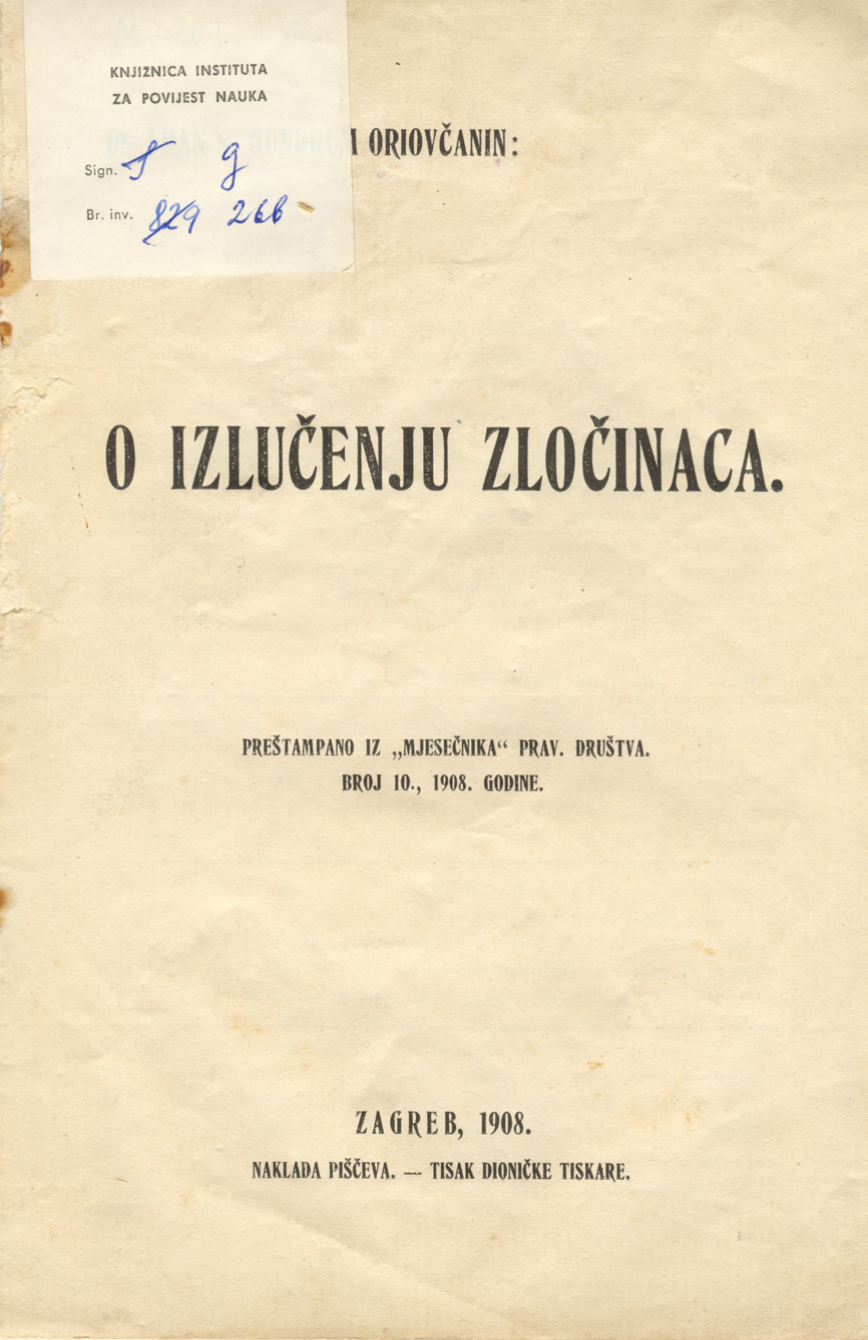
The front cover of the book On Banishment of Criminals (in Croatian: O izlučenju zločinaca), 1908.

Although the problem of criminality did not take as much Gundrum’s attention as prostitution and sexual hygiene, he did dedicate several booklets to this topic. In these texts, Gundrum’s attitudes toward eugenics were more readily expressed than in those dealing with prostitution. On Banishment of Criminals (Figure 5) contains a discussion about healthy and sick criminals, which gradually turns into a discussion about the degenerate (27). Although there is no clear definition of a healthy criminal, we can assume that it refers to personalities who cannot tell the difference between good and evil, although they do not have a psychiatric diagnosis. For such cases, Gundrum suggests either deportation, if the country is big enough or has colonies, or incarceration, if the country is small. On the other hand, there are also sick criminals, whose criminality is caused by a mental disease. These are the criminals who – due to the “current notion of humanitarianism” (27) – have to be treated.

The degenerate are, according to Gundrum, all those who have any congenital defect that interferes with survival. At one point, he writes that the “truth be told, the degenerate are not so much sick, as they are stunted” (27), and then he says that incarceration of criminals would make sense “if some of the degenerate were categorized as healthy (...), but we should not incarcerate the sick” (27). Obviously, Gundrum included both sick and healthy criminals among the degenerate, along with all other degenerate individuals who suffered from congenital defects. It is important to notice that the attribute healthy was for Gundrum a conditional category and applied to those who did not have a predisposing psychopathology for their condition; it did not mean that they were healthy in the absolute sense of the word. Gundrum thought that there was a predisposing factor responsible for incorrigible, corrupt morality even in a healthy criminal. Consequently, he believed that castration of incorrigible criminals and rapists was a method that promised a solution to the problem of criminality (27). Castration, of course, would have made no sense if Gundrum had not assumed that criminal behavior was determined by heredity. Since, thereby, all criminals belonged to the same group as the degenerate, they were all potential candidates for eugenic measures.

Gudrum believed that the degenerate were mostly the result of modern medical advances combined with ethical feeling of empathy: “Science has found the most refined means and ways to achieve this purpose; there are different institutions, which cost a lot of money and where everything is arranged, everything aimed to act against nature, not only to save such a creature, but also to make it somehow capable of reproduction, a creature that nature would know how to dispense of very quickly” (27). All these means, therefore, have one single goal – “to act against nature” (27), which always finds a way to eliminate the degenerate. While “nothing degenerate can be found in nature” (27), because natural selection takes care of it, people do everything they can to save the weak and ill and help them reach fertile age at which they can start spreading their hereditary defects further. Gundrum obviously thought that good intentions had no place in objective medical profession and that society rather than the individual deserved empathy.

Gundrum extended his debate with Lombroso to the subject of criminality. He based his criticisms of Lombroso’s theory about born criminals (31) on statistics, pointing out that only 15 of 2804 people sentenced for crime in 1907 were brought to the Royal Earth Institute for Mentally Ill in Stenjevec (32). After excluding simulants and patients with no diagnosable mental disorders, he found that only 8 had committed a crime in the state of unsound mind. Although Gundrum believed that statistics did not support Lombroso’s theory of born criminals, he still gave him credit for opening the door to research into “physical and mental inferiority of some people” (32). Gundrum’s attitude toward healthy criminals – and health for him was a relative concept – is confirmed by the following words: “(...) I have never intended to claim that all other criminals – 2796 of them – were unconditionally and absolutely mentally healthy; only that they showed no signs that would justify the conclusion that they had a mental disorder (...)” (32). In other words, the nature of hereditary burden in their case was not psychiatric, but moral. However, Gundrum was very cautious and indirect in his critique of Lombroso’s attitudes, saying that his research only provided evidence that Lombroso’s theory “cannot be applied to our circumstances” (32), and left open the possibility that the circumstances in other countries were different and the problem of degeneration possibly more widespread. Irrespective of the fact that the number of mentally ill criminals was low, Gundrum was convinced that it was a big problem that seriously threatened the feeling of safety in communal life, because all crimes could have been prevented: “This should prompt all counties into action to treat the mentally ill who fornicate freely with anyone, because these poor creatures are incapable of comprehending the act that they will eventually commit, while the community teeters steadily on the edge of a dire calamity. In this case too the same old maxim applies – preventing evil takes precedence over everything else!” (32). This issue was not important only from the point of civil safety, but also from the economic point of view: “A large sum of money, I’d say, if we take into account that this money actually serves to achieve a negative gain, that is, to remove everybody sick in their mind, and some of those who are really dangerous. This huge expense and heavy burden on public budget does not have a single positive result. What a sad fact!” (32). Gundrum did not think that huge expenses incurred to the state economy by some individuals were good enough a reason to remove the degenerate from the social body. What distinguished criminals from all other degenerates, whose treatment costs could also be high, was that criminals were dangerous to others. Gundrum was preoccupied by those who presented a threat to the society; financial threat to the society was not a sufficient reason – society had to be threatened by violence.

One of the arguments repeatedly used by Gundrum in the debate about criminals was especially important as it reflected the complexity of his attitudes. Gundrum investigated the role of alcohol in crime on several occasions. We chose two discussions on the topic of alcohol, one from 1904, titled Alcohol-Poison, and the other from 1909, titled Crime and Mental Disease. In Alcohol-Poison, Gundrum presented statistics obtained from the director of the Institute for Mentally Ill in Stenjevec for the period 1893-1902, which clearly showed that a third of mentally ill patients were classified as alcoholics. He also used the statistics from Germany, according to which 41.7% of prisoners were alcoholics. Gundrum’s conclusion was unambiguous: “Thus we can say without hesitation that alcohol creates insanity and criminality” (3). He established a hereditary link between parental alcoholism and criminality of their offspring. One of the mechanisms by which Gundrum thought alcohol promoted criminal behavior was a direct effect of alcohol on hereditary matter, which was considered the main reason why children born to alcoholic parents had alcoholism and criminality “in their blood.” The other was the role of drunkenness in a particular criminal act: “80% of all crimes occur in a state of drunkenness” (3). The latter mechanism equally affected the mentally healthy, “whose mind became deranged due to pleasures of alcohol” (3), and mentally ill individuals, “whose primary illness has worsened due to pleasures of alcohol or who were stimulated to commit a misdeed because of drunkenness (...)” (32). In addition to those who became alcoholics because they inherited it from their parents, Gundrum also recognized the influence of the “man’s desire to imitate” (3).

Gundrum mentioned three methods that could be used to solve the problem of criminality. One was deportation, which was an option for all healthy criminals. To support this attitude, he described the experiment of deportation of “the worst thieves, scapegraces, brigands, and man killers” to Australia (27), where reversion of hereditary burden occurred under the influence of new circumstances and return to nature. After only a few generations, there was “truly a very solid stratum of normal people” (27). This also reflected his attitude that reformation could have been achieved after only a few generations even in those who had already deeply sunk in the life of crime, but only if they were completely deprived of the culture that had created them. In other words, they should have been left to the strong, existential pressure of nature, which did not necessarily and exclusively include natural selection: “If he knew that he was to remain in the new homeland until the end of his life, then, after probably a horrible storm that would arise in his soul at the beginning of incarceration, he would get accustomed to new relations. He would work. He would have to work unless he wanted to perish; the work would sustain him, drive him (...)” (27). Here, the pressure of reform was given precedence over natural selection. Such a pressure could not be created by a society, which was based on the reduction of existential pressure. A single instance that could make such a radical reform was the nature itself. Thus, in this case, we can clearly see that Gundrum understood the deportation method in the Lamarckian context, where nature provided the conditions for inheritance of characteristics acquired over time, such as persistence and engagement.

Two more methods suggested by Gundrum were influenced by the laws passed in two American states, Ohio and Indiana. The first one, which Gundrum considered to be of limited effectiveness, was the Ohio Law on Marriage from 1904, which prohibited marriage to “the mentally ill, idiots, and epileptics” (27). It is interesting that Gundrum omitted “chronic alcoholics” from this list, although they were included in the original Law. The other was the Indiana Law on Sterilization from 1907, which applied to “incorrigible criminals, the slow-witted, feebleminded, and rapists” (27). Obviously, incorrigible criminals (healthy criminals) were the candidates for sterilization, as well as the feebleminded (sick criminals). Such an attitude could have been justified only if Gundrum had thought that healthy criminals also carried a hereditary factor for incorrigible criminality, which was highly probable given the fact that he considered morality to be instinctive, biologically determined. Since criminals incurred high costs and represented danger to the society, in Gundrum’s opinion, the sterilization law was reasonable: “By all means, a Draconian law! – because it is not safe from abuse; and if there were no possibility of it being abused, than everyone would deem this law to be purposeful, because castration today is not a dangerous operation and no one would object it, just as no one objects to laws and regulations on protection from contagious diseases or laws on vaccination against smallpox or against unlimited power of police. Many shall deem this legal novelty scandalous, but every one, even the most cold-blooded skeptic, will have to admit that no radical novelty, however noble, has found its place without practical experiments” (27).

It is evident that the use of carefully chosen analogies with the police and vaccinations served the purpose of marginalizing the side-effects of the absolute power of physicians. Being noble toward criminals has its limits and Gundrum reminded his contemporaries of priorities: “It is good to have patience with these wretches and it is noble to treat them humanely; but when they become dangerous to our neighbor, to mankind, then the most radical means should be used to remove this evil” (27). At the end of his book about criminals, Gundrum fully supports further development of care for the problem of criminals: “We should greet with joy every effort and undertaking by those who work on reducing the evil and eliminating dangerous individuals from the society of mankind” (27).

## Discussion and conclusion

Until now, Gundrum has been presented exclusively as a forerunner of hygienic efforts and health enlightenment in Croatia (1). On the other hand, eugenic aspects that formed the axis of his hygienic efforts have not been analyzed. Although Gundrum did not use the expression “eugenics” in his writings, his works were proven to be full of traces of Darwinian ethics, Lamarckism, and eugenics. There were also clear elements of negative eugenics, upon which Gundrum partly based his concept of social protection.

While he demonstrated moderation when suppression of prostitution was concerned, he completely adopted eugenic measures for the repression of criminality. Among other concerns, Gundrum analyzed the economic aspect of putting into prisons and asylums those who, due to their disease, represented a threat to the society, and believed that such a practice produced only a negative benefit. By negative benefit he meant the exclusion of a particular group of people from the society, the act which by itself did not produce a new value or increased the benefit for the society. A positive benefit would include the process of building a society, which would bring an additional value to the society without criminals. Even in such attitudes, we can catch glimpses of a certain divergence from the curative approach and recognize the rudiments of the idea that not only somatic, but also social pathology, can be prevented. It is evident that Gundrum was more interested in the safety of society as a whole and less interested in the rights of individuals. The society, if threatened by its sick members, has the right not only to protect itself, but also to do it in the cheapest way, financially and otherwise.

Gundrum’s writings, ideas, and attitudes were occasionally inconsistent or even contradictory. A good example is his ambivalent attitude toward the concept of fight for survival. On the one hand, deportation of criminals to Australia reflected the Lamarckian view that fight for survival underlies the reformation of individuals. On the other hand, when he analyzed the consequences of life in a city a year later, Gundrum concluded that “the percentage [of mentally ill] is high there where the fight for survival is most violent, where physical and mental health care is most defective (...)” (32). In villages, the percentage of mental diseases was lower, because living conditions were simpler and “fight for survival was not so bitter, so brutal (...)” (32). While the fight for survival in Australia quickly created a whole generation of normal people from the worst individuals, the same fight for survival in the cities produced quite the opposite effect. It is unclear whether Gundrum was aware of this contradiction in his attitudes and whether he completely understood the mechanisms of the fight for survival, which had positive effects in Australia and completely opposite effects in Western European cities.

The question is how we should explain Gundrum’s advocacy of sterilization, when he was clearly aware that deportation was an effective method for reformation of the worst individuals. The deportation method was in line with social Darwinism, which presupposed cancellation of all achievements of civilization that make people weak. On the other hand, sterilization was a method advocated by those who favored eugenics, who believed that civilization would be preserved if artificial selection was applied on the level of society (13). Basically, Gundrum believed that the main criterion in choosing between two methods was feasibility. Thus, in case of countries without colonies to deport their criminals to, the method of choice was negative eugenics (sterilization), which Gundrum considered to be cheap and safe (27); otherwise, deportation should be favored.

Although there was a strong correlation between alcoholism and criminality in Gundrum’s opinion, he still did not suggest sterilization for alcoholics. Alcoholism as an important social trigger of criminality was still not considered a condition serious enough to declare the individuals addicted to alcohol as a threat to the society. Although they were capable of criminal acts, and most criminal acts were committed under the influence of alcohol, not many alcoholics actually committed a crime. Therefore, it follows that only a social threat, which could have been established with certainty only in criminals and mentally ill, could justify the use of radical methods. In addition, alcoholism was, in practical terms, such a wide problem that Gundrum did not see sterilization of alcoholics as a reasonable approach.

Created in times when social processes were interpreted and understood in the context of anthropological, organicistic approach, Gundrum’s work reflects the shift in the focus of medicine from the illness of an individual to the illness of a society. Criticism of medicine as a profession that does everything in its power to save those who do not belong to the society only apparently undermined Gundrum’s own position. His concept of public health and medicine, increasingly adopted by other physicians at the time, included a strong state control and repression of the sick in order to preserve the healthy social body. Medicine was supposed to be primarily preventative rather than curative profession, including the prevention of inheriting bad characteristics. Fortune or misfortune was not reflected at an individual level, but at the level of a society, and society had to do everything it could to ensure its happiness, even if it meant removing inadequate elements from the social body. Of course, except for the aberrant eugenic movement, there were some very useful humanistic ideas that appeared with new understanding of the role of medicine. This was particularly obvious in case of prostitution, where the binomial illness/poverty marked the beginnings of the ideas that would become more prominent in the works of representatives of social medicine movement, especially Andrija Štampar. Identifying the poor as ill paved the way for improvement of health by introducing broad social changes.
